# AutoCoV: tracking the early spread of COVID-19 in terms of the spatial and temporal patterns from embedding space by K-mer based deep learning

**DOI:** 10.1186/s12859-022-04679-x

**Published:** 2022-04-25

**Authors:** Inyoung Sung, Sangseon Lee, Minwoo Pak, Yunyol Shin, Sun Kim

**Affiliations:** 1 Interdisciplinary Program in Bioinformatics, Seoul National University, Seoul, Republic of Korea; 2 Institute of Computer Technology, Seoul National University, Seoul, Republic of Korea; 3 Department of Computer Science and Engineering, Seoul National University, Seoul, Republic of Korea; 4 Interdisciplinary Program in Artificial Intelligence, Seoul National University, Seoul, Republic of Korea; 5 AIGENDRUG Co., Ltd., Seoul, Republic of Korea

**Keywords:** COVID-19, SARS-CoV-2, Deep learning, Sequence embedding, Early spreading pattern

## Abstract

**Background:**

The widely spreading coronavirus disease (COVID-19) has three major spreading properties: pathogenic mutations, spatial, and temporal propagation patterns. We know the spread of the virus geographically and temporally in terms of statistics, i.e., the number of patients. However, we are yet to understand the spread at the level of individual patients. As of March 2021, COVID-19 is wide-spread all over the world with new genetic variants. One important question is to track the early spreading patterns of COVID-19 until the virus has got spread all over the world.

**Results:**

In this work, we proposed AutoCoV, a deep learning method with multiple loss object, that can track the early spread of COVID-19 in terms of spatial and temporal patterns until the disease is fully spread over the world in July 2020. Performances in learning spatial or temporal patterns were measured with two clustering measures and one classification measure. For annotated SARS-CoV-2 sequences from the National Center for Biotechnology Information (NCBI), AutoCoV outperformed seven baseline methods in our experiments for learning either spatial or temporal patterns. For spatial patterns, AutoCoV had at least 1.7-fold higher clustering performances and an F1 score of 88.1%. For temporal patterns, AutoCoV had at least 1.6-fold higher clustering performances and an F1 score of 76.1%. Furthermore, AutoCoV demonstrated the robustness of the embedding space with an independent dataset, Global Initiative for Sharing All Influenza Data (GISAID).

**Conclusions:**

In summary, AutoCoV learns geographic and temporal spreading patterns successfully in experiments on NCBI and GISAID datasets and is the first of its kind that learns virus spreading patterns from the genome sequences, to the best of our knowledge. We expect that this type of embedding method will be helpful in characterizing fast-evolving pandemics.

## Background

In December 2019, a new virus, severe acute respiratory syndrome coronavirus 2 (SARS-CoV-2) broke out as coronavirus disease 2019 (COVID-19), and in March 2020, the World Health Organization (WHO) declared a pandemic [1–3]. Various vaccines are being developed as a result of the efforts of numerous scientists to overcome COVID-19, but the effectiveness of vaccines against the newly occurring mutations is not well established yet [4–6]. As of August 2020, more than 21 million people were infected with SARS-CoV-2 across the world. In the United States, with the largest number of infections, 5.23 million or 1.7% of the U.S. population were infected [7]. As the virus has spread, the number of sequenced SARS-CoV-2 genomes increased and the sequence has been analyzed mainly for point mutations. Mutation-based studies classified the class of SARS-CoV-2 according to the type of variants and reported that COVID-19 in early Asia and Europe were caused by different types of viruses [8, 9]. In addition, the basic reproductive number ($R_0$), which indicates the degree of transmission of the disease, showed that different types of variants had different rates of spread [10]. As of March 2021, COVID-19 is wide-spread all over the world with new genetic variants. Once the virus becomes globally widespread, it is very difficult to track the spreading patterns of COVID-19 because of external factors such as the global lockdown and vaccinations. However, while the virus is being spread, i.e., early stage of a disease epidemic, it might be possible to track spreading patterns in terms of spatial and temporal characteristics.

According to Nextstrain’s August 2020 SARS-CoV-2 situation1 reports, the virus has mutated over time as the virus spreads to different regions [11]. Different strains of the virus evolve as the virus spreads to different regions over time (see the Additional file 1: Fig. S2). Based on the observations, there is no doubt that SARS-CoV-2 genome sequences have different characteristics depending on regions and time but are not yet fully understood. In this paper, we define such unknown information in terms of spatial patterns and temporal patterns, respectively. Thus, understanding the spatial and temporal characteristics of virus spreading patterns is a very important task. However, our knowledge so far is limited to simply measuring how many patients have occurred geographically and temporally. Beyond the simple statistics, the spread at the level of individual patients can be investigated through an embedding space with spatial and temporal features.

**Fig. 1:**
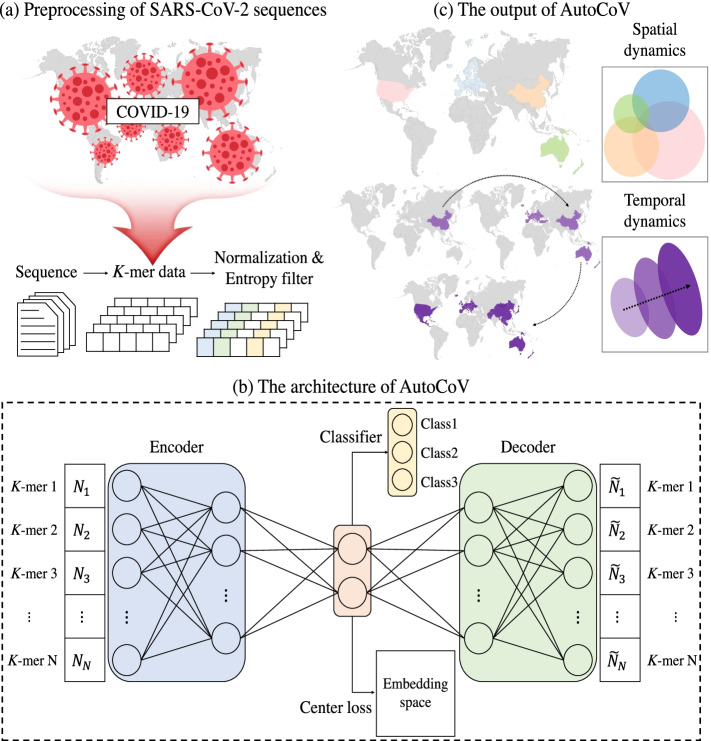
The overall framework of the AutoCoV model. **a** Preprocessing of SARS-CoV-2 sequences: We transform the virus sequences into a *k*-mer vector. After frequency normalization and information theory based *k*-mer filtering, we obtain an informative *k*-mer frequency matrix as inputs for AutoCoV. **b** The architecture of AutoCoV: It consists of three modules for learning the spatial and temporal patterns of SARS-CoV-2. Auto-Encoder Network generates latent representations that reconstruct the input matrix. Classifier Network guides the latent representations to identify the spatial and temporal patterns. Center Loss module complements the Classifier Network to create a more dense and well-separated embedding space. **c** The output of AutoCoV: The embedding space generated by AutoCoV aims to imply the spatial and temporal patterns of SARS-CoV-2

The main goal of our study is to investigate the tracking of COVID-19 in terms of biological perspectives. When external factors such as global lockdown and vaccination are enforced, it is difficult to investigate the spreading potentials of COVID-19 per se, thus we analyzed the early spread pattern of COVID-19 using the SARS-CoV-2 sequences up to July 2020. In this work, we propose a deep learning method, AutoCoV that can track the early spread of COVID-19 (Fig. 1). Tracking the virus spreading patterns of spatial and temporal characteristics was achieved by an auto-encoder based latent representation deep learning model. Since there are quite a number of mutations in a virus sequence, a sequence is preprocessed using *k*-mer, also known as *n*-gram in natural language processing for information theoretic filtering. Then, the input to AutoCoV is a set of informative *k*-mers for a virus sequence with spatial or temporal information. By augmenting an auxiliary classifier and a center loss objective function on the auto-encoder, we guided the latent representation to learn the spatial and temporal patterns. Formally, AutoCoV optimizes three objective functions, such as reconstruction loss, classification loss, and center loss, as shown in Eq. 9. In this paper, we used SARS-CoV-2 sequences from two different datasets: the National Center for Biotechnology Information (NCBI) and the Global Initiative for Sharing All Influenza Data (GISAID) [12–14]. As a result, we showed that our model outperformed baselines on an experiment of annotated SARS-CoV-2 sequences in the NCBI dataset. An extensive ablation study showed the contributions of each component and strategy used in AutoCoV. Furthermore, we demonstrated the robustness of the embedding space generated by AutoCoV using the NCBI dataset against an independent GISAID dataset.

### Related works

Since the first outbreak of the COVID-19 virus in 2019, numerous papers have been published. Early papers conducted biological evolutionary and structure analysis of SARS-CoV-2 [15, 16]. As the number of SARS-CoV-2 genome sequences increased, mutation-based research was conducted and cluster analysis was performed on sequences with the same mutation [17, 18]. Some papers statistically summarized spatial and temporal features according to the types of mutations [9, 19, 20]. Recently, many studies have been conducted using machine learning technologies, for examples, diagnosing COVID-19 using medical images by applying convolutional neural network (CNN) [21–23], predicting antiviral drugs using natural language processing (NLP) [24], or using deep neural network (DNN) [25] to form a drug repurposing perspective. In addition, research on the current outbreak status and spread of COVID-19 using demographic or mobility data [26, 27], and the impact of external factors such as global lockdown policies were also studied [28, 29]. However, there have been no studies on the spatial and temporal spread of COVID-19 utilizing the SARS-CoV-2 sequence.

Analyzing long biological sequences at the character level, i.e. single resolution, is a very difficult task. Although single nucleotide-based analysis may utilize more rich information about the sequences, large amounts of features may lead to the curse of dimensionality, especially in the long length of biological sequences. Therefore, it is important to use an appropriate encoding method that can reflect biological meaning while reducing the dimension of the sequence. There are simple but widely used methods of expressing the characteristics of the sequences such as one-hot encoding or *k*-mer encoding. One-hot encoding of the sequence data is straightforward and it has the advantage of preserving the positional information of the sequence, whereas the encoding length depends on the length of the sequence [30, 31]. *K*-mer encoding, on the other hand, loses positional information, but it has the same encoding length for a fixed value of *k*, and has the advantage of being able to learn the local context of a biological sequence [32–35]. In the case of SARS-CoV-2 genome sequences, the sequences are very similar to each other both in length and of nucleotide compositions and only a small number of variants determine the pathogenic properties of the virus. There is also a study on the possibility of additional mutations occurring around mutations [36]. Therefore, in this study, we used a *k*-mer based approach to encode the sequence to focus on the local context of the sequence rather than the location information.

There are many existing machine learning algorithms for biological sequences that utilize the *k*-mer information as input features of models in various ways and learn embedding vectors of the *k*-mers or the sequence itself. For example, word2vec [37] or doc2vec [38] based models were proposed such as BioVec [37], dna2vec [39], or Super2Vec [40]. Hybrid approaches of CNN and recurrent neural network (RNN) were also proposed to capture local contexts of *k*-mers and long-range interactions within the sequence [41, 42]. However, most existing methods are designed for short-length of biological sequences and these methods have difficulty in learning embedding vectors of long-length sequences such as SARS-CoV-2.

**Table 1:** The summary of datasets (as of 2020-07-17)

	NCBI (5,210)	GISAID w/o NCBI (61,210)
Subclass	S (1,246), L (266), V (130), G (463), GR (418), GH (2,687)	S (4,328), L (3,856), V (4,418), G (14,982), GR (19,316), GH (14,310)
Spatial	Asia (454), Oceania (403), Europe (280), North America (4,073)	Asia (3,805), Oceania (2,151), Europe (41,365), North America (13,889)
Temporal	Early (178), Middle (2,632), Late (2,400)	Early (1,175), Middle (25,058), Late (34,977)

Each dataset has three categories of SARS-CoV-2 characteristics: Pathogenic mutations (Subclass), Spatial, Temporal. The value in the parenthesis denotes the number of sequences. The detailed information about Subclass label was described in Additional file 1: Table S1

## Results

### Dataset

SARS-CoV-2 sequences were obtained from two different databases: NCBI Virus [43–45] (http://www.ncbi.nlm.nih.gov/genome/viruses/) and GISAID (http://www.gisaid.org). From NCBI Virus, we collected 7,031 sequences including SARS-CoV-2 reference sequence NC_045512, as of July 17, 2020. NCBI Virus sequences had spatial and temporal labels based on collected continent and date. And each sequence was fully annotated with information about the location of the genes. From GISAID, we collected 61,210 sequences, as of July 17, 2020. GISAID sequences had spatial and temporal labels as well as subclass labels based on pathogenic mutations defined by the GISAID nomenclature system.

**Fig. 2:**
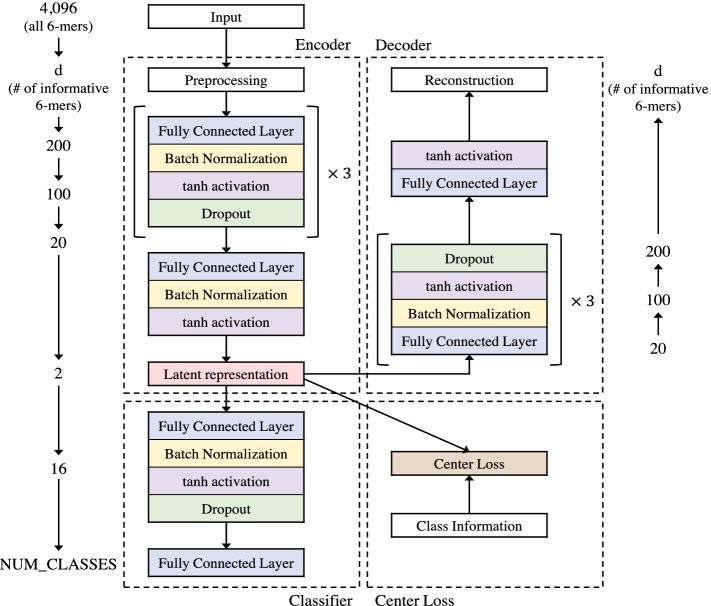
The structure of AutoCoV. It consists of three modules: auto-encoder, classifier, and center loss. For each layer, the number of neurons is shown beside the corresponding layer

### Experimental setup

For input data of the model, we used 5210 sequences commonly contained in NCBI Virus and GISAID. Each sequence was about 30,000 bases in length and had spatial and temporal labels. For the spatial label, the sequences were divided into four classes based on the collected continents: ‘Asia’, ‘Oceania’, ‘Europe’, and ‘North America’. For the temporal label, the sequences were divided into three classes based on March 2020, when the spike protein mutation occurred [8, 9]. Based on March 2020, when the D614G mutation in which Aspartic acid (D) at position 614 of spike protein mutated to Glycine (G) occurred, the sequence before March 2020 in which there was no D614G mutation is labeled as ‘Early’ and the sequence in March 2020 in which the D614G mutation occurred is labeled as ‘Middle’. And the sequences after March 2020, when the D614G mutation has spread around the world, are labeled as ‘Late’. Mutations in non-protein-coding regions were also important [46, 47], but due to different sequencing techniques and sequencing lengths of each sequence, there were lost in some sequences. Thus, only the protein-coding regions from ORF1ab to the N gene were used for quality control of the sequence. Among the sequences only from GISAID, we excluded low-quality sequences such as sequences with lowercase nucleotide or more than 100 N’s, so we used 23,979 as an external dataset to test the robustness of the embedding space learned from the NCBI dataset (Detailed GISAID dataset preprocessing is described in Additional file 1). The details of datasets are listed in Table 1. We split NCBI dataset into training, validation, test data split with 80:10:10 ratio with preserving the spatial/temporal label ratio and performed the stratified split 10 times. In the case of learning spatial patterns, class labels are ‘Asia’, ‘Oceania’, ‘Europe’, and ‘North America’ for supervised learning. Similarly, in the case of learning temporal patterns, class labels are ‘Early’, ‘Middle’, and ‘Late’. All reported figures and tables in this section were created using the test data only. Detailed structures and hyper-parameters of models are described in Fig. 2 and Additional file 1.

#### Baseline methods

The performances of AutoCoV on learning spatial and temporal patterns were compared with seven baselines belonging to the three categories: ‘Dimension Reduction’, ‘Unsupervised’, and ‘Supervised’. In case of ‘Dimension Reduction’, three conventional dimension reduction techniques were used such as Principal Component Analysis (PCA) [48], t-Stochastic Neighbor Embedding (t-SNE) [49], and Uniform Manifold Approximation and Projection (UMAP) [50]. The input data of the three methods was the same *k*-mer frequency matrix as AutoCoV. In case of ‘Unsupervised’, two sequence embedding methods such as dna2vec [51] and seq2vec [39] were utilized. In the case of ‘Supervised’, two well-known deep learning models for nature language processing were utilized with augmentation of a classifier network (CF), which was similar with AutoCoV: Seq2Seq+CF [52] and BERT+CF [53]. In the contrast with ‘Dimension Reduction’, the baselines of ‘Unsupervised’ and ‘Supervised’ used the raw genome sequences of SARS-CoV-2 as the input data. Details are described in the Additional file 1.

#### Evaluation metrics

Learning the spatial or temporal patterns of SARS-CoV-2 means that in the embedding space generated by the model, sequences of the same labels are well clustered and sequences of different labels are well separated while well predicting labels of the sequences. Given the spatial or temporal labels of the sequence as ground truth, we used two clustering metrics and one classification metric to measure how well the model learn spatial or temporal pattern in embedding space. The first clustering metric is label homogeneous score (LHS) that measures the purity of class labels within the cluster [54]: $LHS(Y; {\tilde{Y}})$ where *Y* is true class labels and ${\tilde{Y}}$ is predicted class labels by assigned the cluster in the space. The other metric is mutual information score (MI) that measures the dependence between target patterns and axes ($\text {Dim}1\text { and }\text {Dim}2$) of 2D embedding space: $I(Y;\text {Dim}1, \text {Dim}2)$. Lastly, the classification metric is the F$_1$ score of K-Nearest Neighbors classifier (neighbors = 10) where the neighbors are the sequences in the train data. All three metrics are ranged from 0.0 to 1.0 and the higher values represent the good performance. Details about the metrics are described in the Additional file 1.

**Table 2:** Performance comparison results (mean ± std)

Method		LHS	MI	F1
Spatial				
Dimension Reduction	PCA	0.280 ± 0.040	0.357 ± 0.050	0.739 ± 0.012
	t-SNE	0.215 ± 0.043	0.226 ± 0.034	0.690 ± 0.017
	UMAP	0.237 ± 0.030	0.448 ± 0.031	0.837 ± 0.011
Unsupervised	dna2vec	0.204 ± 0.035	0.108 ± 0.046	0.736 ± 0.019
	seq2vec	0.149 ± 0.041	0.238 ± 0.030	0.689 ± 0.018
Supervised	Seq2Seq+CF	0.131 ± 0.030	0.099 ± 0.013	0.689 ± 0.017
	BERT+CF	0.155 ± 0.054	0.327 ± 0.034	0.734 ± 0.013
	**AutoCoV**	**0.529** ± **0.051**	**0.773** ± **0.045**	**0.881** ± **0.012**

Method		LHS	MI	F1
Temporal				
Dimension Reduction	PCA	0.147 ± 0.065	0.198 ± 0.040	0.665 ± 0.024
	t-SNE	0.140 ± 0.052	0.184 ± 0.033	0.384 ± 0.031
	UMAP	0.146 ± 0.053	0.329 ± 0.047	0.671 ± 0.027
Unsupervised	dna2vec	0.117 ± 0.053	0.087 ± 0.030	0.616 ± 0.021
	seq2vec	0.111 ± 0.048	0.156 ± 0.018	0.464 ± 0.015
Supervised	Seq2Seq+CF	0.116 ± 0.066	0.075 ± 0.013	0.474 ± 0.027
	BERT+CF	0.091 ± 0.058	0.181 ± 0.048	0.559 ± 0.037
	**AutoCoV**	**0.355** ± **0.067**	**0.554** ± **0.065**	**0.761** ± **0.023**

Three dimensional reduction methods (PCA, t-SNE, UMAP), two unsupervised methods (dna2vec, seq2vec), and three supervised methods (Seq2Seq+CF, BERT+CF, AutoCoV) are compared, and the bold values represent the best performance among them. In both patterns, AutoCoV outperforms the baselines in all metrics

### Performance comparison of learning embedding spaces

**Fig. 3:**
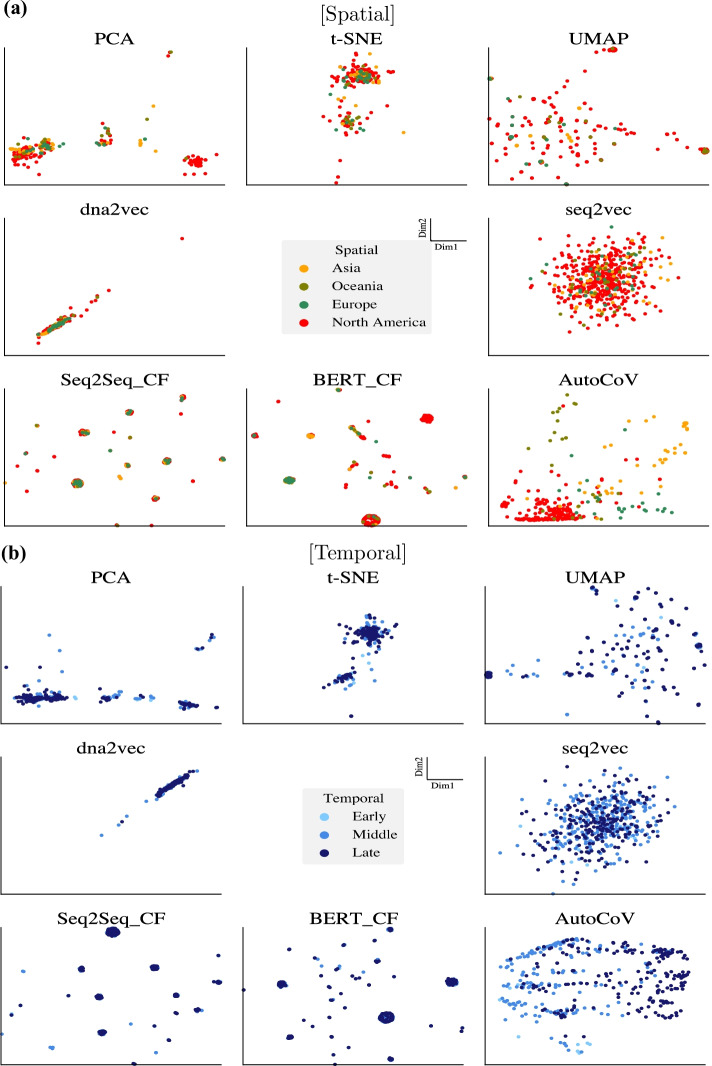
Comparison of spreading pattern visualizations. 2D embedding spaces of baselines and AutoCoV are illustrated on **a** the spatial patterns and **b** the temporal patterns. The data fold with the median performance out of 10 folds was used as input to AutoCoV for the figures. The axes of each figure are set by the axes of training data

We conducted experiments for learning spatial and temporal patterns of SARS-CoV-2. As qualitative results, Fig. 3 shows 2D embedding spaces of SARS-CoV-2 in the view of each pattern. As quantitative results, Table 2 provides the results of the three metrics on each embedding space. From both results, AutoCoV outperformed the baselines in terms of learning the spreading patterns of the virus. We further investigated AutoCoV performance with two additional experiments: effects of different *k*-mer sizes and ablation study. From comparative experiments on varying lengths of *k* ranging from 1 to 7, we showed that when *k* is 6, it can cover the performance aspect of the model and the aspect of biological prior knowledge. Furthermore, in an ablation experiment to measure the contribution of each component of AutoCoV, we showed that AutoCoV, when all components were incorporated, has the best performance. Details of the two additional experiments are in the Additional file 1.

#### Visualization of spatial and temporal patterns

**Fig. 4:**
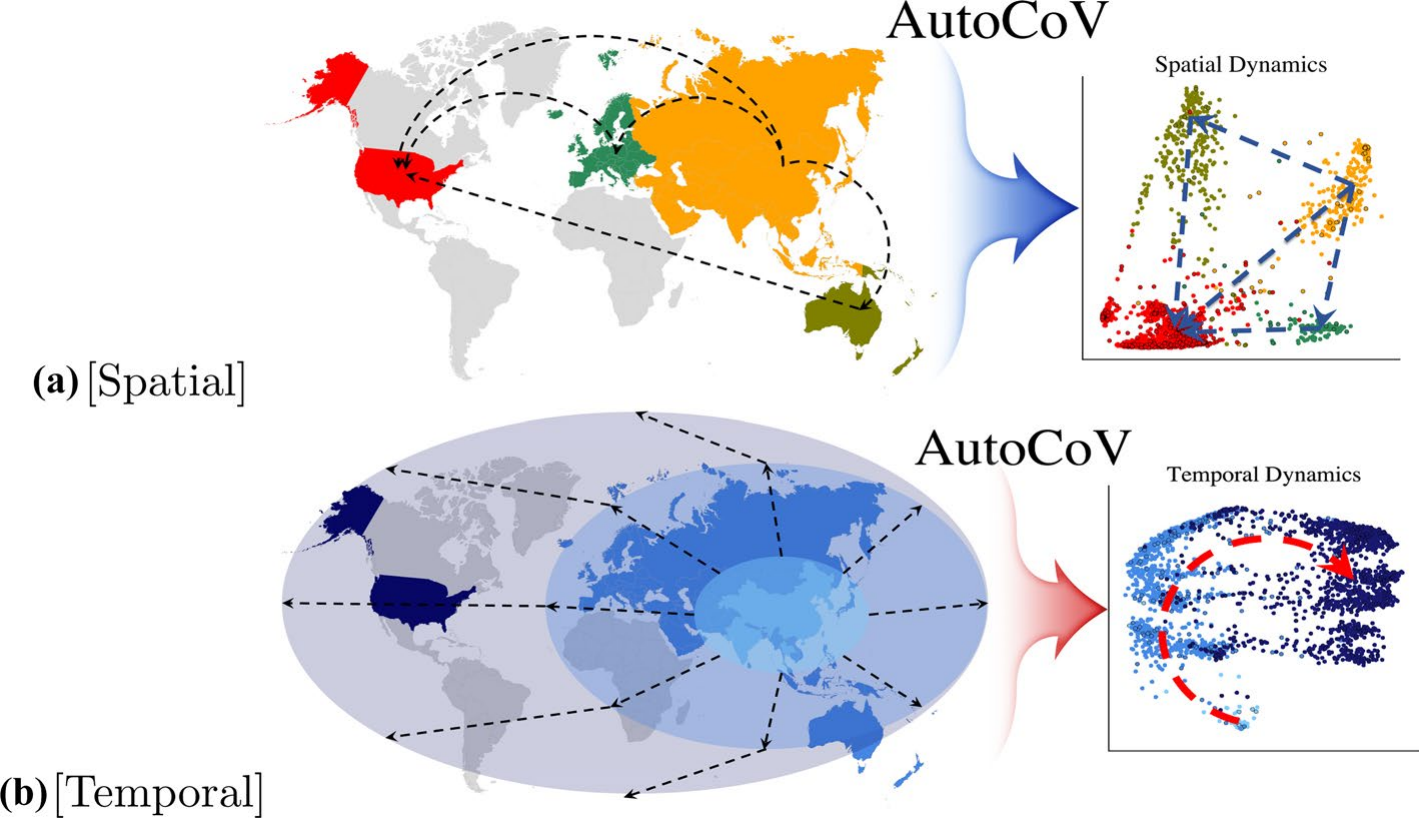
Spreading patterns on each embedding space by AutoCoV: **a** Spatial and **b** temporal. Solid dots represent train data and black-edged dots represent test data

For learning an embedding space representing spatial patterns, AutoCoV constructed the space that successfully distinguishes Asia and North America (Fig. 3a and Additional file 1: Figs. S3(a)–(b) and S4(a)–(b)). Interestingly, the AutoCoV was the only one that could infer the spatial spread of SARS-CoV-2. Figure 4a showed that viruses were well-separated and well-clustered according to continents in the AutoCoV spatial embedding space, and the spread pattern of the virus from Asia through Europe and Oceania to North America could be inferred through the embedding space. This trend was similar to the current outbreak of SARS-CoV-2 until August, and it was consistent with the August 2020 report of NextStrain [11]. In addition, without using subclass labels, AutoCoV also learned subclass characteristics simultaneously (Additional file 1: Fig. S5a). Among the baselines, UMAP showed reasonable spatial patterns, but the same regions were not well clustered. The other baselines showed results with all regions being mixed.

For learning an embedding space representing temporal patterns, AutoCoV distinguished middle and late time points of SARS-CoV-2 (Fig. 3b and Additional file 1: Figs. S3c–d and S4c–d). Furthermore, temporal spreading patterns of SARS-CoV-2 were only observed in the embedding space of AutoCoV. Figure 4b showed that the time points were well-separated in the AutoCoV temporal embedding space, and the temporal spread patterns of the virus could be inferred from the bottom center to the left and then to the right. Likewise, with learning spatial patterns, AutoCoV learned subclass characteristics simultaneously without subclass labels (Additional file 1: Fig. S5b). Among the baselines, dna2vec showed a similar time trend but most of the sequences were extremely tightly clustered, which can hardly be interpreted as the spreading patterns of the virus. Again, the other baselines showed results such as all-time points being mixed or projecting test data into the wrong time points (e.g. t-SNE).

#### Quantitative measurements

AutoCoV outperformed the baselines in all three metrics on both spatial and temporal patterns (Table 2). In the context of spatial patterns, AutoCoV had 2.2 times, 1.7 times, and 4.4% improvements in LHS, MI, and F$_1$ over the best baseline UMAP, respectively. In the context of temporal patterns, AutoCoV had 2.4 times, 1.7 times, and 9.0% improvements in LHS, MI, and F$_1$ over the best baseline UMAP, respectively. Among the baselines, dimension reduction techniques showed better performances than unsupervised and supervised learning methods. This is because the dimension reduction techniques captured local and global structures in both patterns and used *k*-mer frequency matrix as input, like AutoCoV. For unsupervised and supervised learning that used raw sequences as input, rich information was available but it may contain redundant information. Since most of the characters in the SARS-CoV-2 sequences were similar between the sequences, most of the rich information was uninformative and would result in the curse of dimensionality that hinders entire learning processes.

**Fig. 5:**
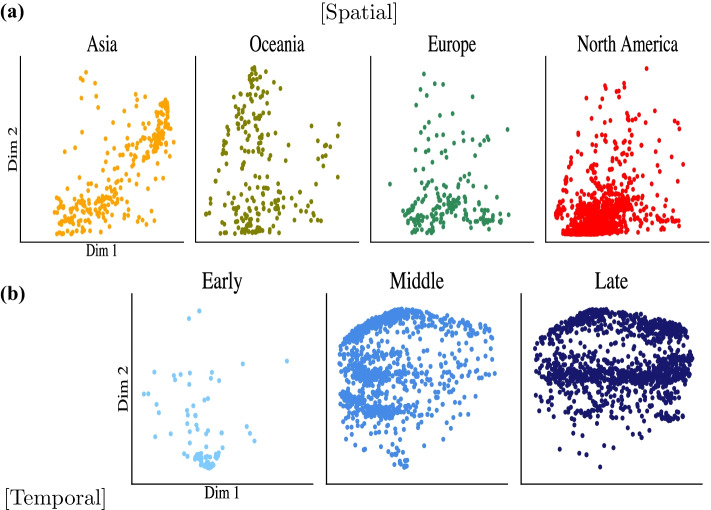
Spreading patterns on each embedding space by AutoCoV in GISAID dataset: **a** Spatial ($0.813 \pm 0.004$) and **b** Temporal ($0.739 \pm 0.005$). F$_1$ score of KNN is described in the parenthesis (mean ± std)

### External dataset validation

To demonstrate the robustness and usefulness of AutoCoV, unannotated sequences in the GISAID as an external dataset, were mapped into the embedding space constructed from the NCBI dataset. To preserve the ratio of each label in the spatial and temporal classes, we performed the stratified sampling 10 times from the GISAID. Details on processing the GISAID dataset are in the Additional file 1. Figure 5 shows the spatial and temporal embedding space results of the fold with median performances in the GISAID dataset. For spatial patterns, the F$_1$ score was about $0.813 \pm 0.004$, and all continents were represented reasonably well in the embedding space (Fig. 5a). All continents spread more widely than those in the NCBI dataset, but this seemed to indicate the influx of mutant viruses by intercontinental migration, as mentioned in NextStrain [11]. For the temporal patterns, the F$_1$ score was about $0.739 \pm 0.005$, slightly lower than the spatial case, but it reflected the time flow of the virus relatively well (Fig. 5b). These results are expected to help characterize fast-evolving pandemics in spatial and temporal embedding space via AutoCoV.

## Discussion and conclusion

### Effectiveness of encoding schemes of SARS-CoV-2 sequences using *k*-mer

In general, supervised learning methods outperform unsupervised learning methods. Interestingly, however, the supervised learning methods (Seq2Seq+CF, BERT+CF) performed even worse than the unsupervised learning methods (dna2vec, seq2vec) or dimension reduction methods (PCA, t-SNE, UMAP) in most LHS, MI, and F1 evaluations on both patterns. On the other hand, AutoCoV performed significantly better than unsupervised methods. We discuss why this happened in terms of the effectiveness of encoding schemes of SARS-CoV-2 sequences using *k*-mer.

AutoCoV extracted *k*-mer information from the sequence and transformed one sequence as a *k*-mer frequency vector with entropy filtering. In the AutoCoV encoding scheme, positional information of *k*-mers was lost. Meanwhile, Seq2Seq+CF and BERT+CF preserved the sentence structure with positional information of *k*-mers. Intuitively, keeping the positional information of *k*-mers seems more natural for biological sequences, but there is a good reason why the AutoCoV encoding scheme performed better. SARS-CoV-2 sequences are very similar to each other, and a small number of variants determine the pathogenic properties of the virus. The order of *k*-mers in the sequence is nearly identical, so it is not useful for distinguishing between SARS-CoV-2 sequences. Therefore, AutoCoV did not utilize the positional information and focused on *k*-mer count changes of the informative variants.

#### Limitations and further studies

In experiments on NCBI and GISAID datasets, it was shown that AutoCoV can effectively model spatial or temporal patterns in the SARS-CoV-2 sequence. There are a number of limitations in our approach. While the *k*-mer encoding scheme of AutoCoV works well for SARS-CoV-2 sequences, this scheme may lose positional information. We can interpret which *k*-mers are important, but it is hard to know exactly where the *k*-mer is in the sequence. Therefore, analysis through additional learning is required to know the exact location of the important *k*-mer in the sequence. In addition, more find-grained information has been lost because temporal labels were discretized in the experimental setup. There may also be sampling bias issues because the amount of data for each label was not adjusted to reflect the prevalence of diffusion.

To compensate for these shortcomings, future studies include improving the interpretability of the model by adopting attention mechanisms [55] or analyzing saliency map of *k*-mer vectors like compute vision domain [56]. In addition, utilizing denoising techniques will enhance the robustness of the model and mitigate the effects of sequence noises. It also seems possible to improve model structures so that more fine-grained label information can be modeled in the embedding space using continuous label representation and unbiased manipulation of the label data.

#### Conclusion

In this paper, we proposed AutoCoV that learned 2D embedding spaces for modeling the spatial and temporal patterns in the early spread of COVID-19. To the best of our knowledge, AutoCoV is the first of its kind that learns virus spreading patterns from the genome sequences. The technical contribution of this paper is that AutoCoV can effectively handle the long-length SARS-CoV-2 genome sequences using information theory and auto-encoder based deep learning model. The biological significance of AutoCoV is the ability to map SARS-CoV-2 genome sequences to 2D spaces that preserve the spatial and temporal patterns without knowing subclass information of the SARS-CoV-2 genome sequences. In extensive experiments, AutoCoV outperformed current embedding spaces learning and visualization methods. The generalization power of the embedding space was demonstrated in a cross validation experiment using the NCBI dataset and in another experiment with an independent GISAID dataset. The main contribution of our work is that a machine learning approach is shown to be capable of learning the spread of COVID-19. We expect that the embedding space that can reflect temporal and spatial features can potentially be useful to track spread dynamics between sequences.

In closing this paper, we emphasize the importance and significance of learning the sequence embedding space for spatial and temporal patterns of the virus spread. A virus evolves into new types often with more aggressive characteristics even in the quarantined countries. Furthermore, infection among people can make different types of viruses, which makes the understanding of virus evolution more complicated. As of now, we do not have computational methods to model these complex spreading patterns spatially and temporally from the virus genome sequences. Existing studies have mainly studied virus propagation by external control factors such as lockout policies based on demographic data or mobility data [28, 29, 57]. As we showed in this study, methods for embedding or representation learning have potential for this important but challenging problem. We expect that comprehensive analysis of statistical methods based on demographic data and this type of embedding method will help characterize the rapidly evolving pandemics.

## Methods

AutoCoV consists of four modules: *Sequence Preprocessing, Auto-Encoder Network, Classifier Network, and Center Loss* for learning spatial and temporal patterns of SARS-CoV-2 sequences by constructing two dimensional (2D) embedding spaces.

### Sequence preprocessing

Let $\mathbf{X }$ denote a $n \times d$ matrix where *n* is the number of sequences and *d* is the number of *k*-mers, $d = 4^k$. In this work, we use $k=6$ for learning embedding spaces of the sequences by *k*-mer approach, which can reflect the biological prior knowledge such as dicodon, i.e., two amino acids. Of course, 3-mers in the DNA sequence are also useful because they can be considered codons, but the number of features that can be expressed as 3-mers is limited than 6-mers. For this reason, $d = 4^6 = 4,096$ is used for the input matrix $\mathbf{X }$. Because most of the sequences are almost identical except for a few character mismatches, frequencies of most *k*-mers are the same and uninformative. An information theory-based *k*-mer feature selection process is performed to distinguish subtly different sequences and to reduce the number of features. On each *k*-mer, an entropy value $H(\cdot )$ is calculated to measure the variance of $\mathbf{X }_{*j}$, i.e.,

1 $$\begin{aligned} H(X_{*j}) = - \sum _{m=1}^{M_j} p(\phi _m)\, \text {log }p(\phi _m) \end{aligned}$$

where $\mathbf{X }_{*j} = [ x_{1j} \; x_{2j} \dots x_{nj} ]^T$ denotes the *j*th column vector of the input matrix $\mathbf{X }$, $M_j$ denotes the number of observed frequency count differently in $\mathbf{X }_{*j}$, i.e., $M_j = |\text {Set}(\mathbf{X }_{*j})|$ , $\phi _m$ denotes the observed frequency count in $(\text {Set}(\mathbf{X }_{*j})$) and $p(\phi _m)$ denotes the probability of observation of $\phi _m$ in $\mathbf{X }_{*j}$. As various *k*-mer frequency counts are observed, the entropy value increases. It means that the *k*-mer is likely to contribute to distinguishing the sequences. Using the entropy value $H(\mathbf{X }_{*j})$ of each *k*-mer, we exclude uninformative *k*-mers that have entropy values smaller than 0.2, an empirically determined threshold. Using the cutoff, *k*-mers that show the same count in 99% of the entire sequences are discarded. Thus, the input matrix *X* is reduced to the smaller matrix $\mathbf{X }'$, a $n \times d'$ matrix where $d'$ is the number of informative *k*-mers by the information theoretic filtering.

After the *k*-mer filtering step, two-level normalization steps are performed: sequence-level and *k*-mer feature-level. We generated a *k*-mer vector of each sequence using protein-coding regions. However, there are slight differences in length for each sequence due to problems such as differences in sequencing techniques. To reduce the sequence-length dependency, as sequence-level normalization, the frequency count of a *k*-mer is divided by the total frequency count of the informative *k*-mers. After sequence-level normalization, the *k*-mer feature level normalization is done by the standardization on each *k*-mer feature. We used StandardScaler from scikit-learn library of Python. Finally, for the next deep learning modules, *tanh* activation function is applied to the normalized input matrix to adjust the scale of each *k*-mer feature. We denote the result of the preprocessing module as $\mathbf{X }_{norm}$.

### Auto-encoder network

We use an Auto-Encoder Network in AutoCoV to learn latent representations $\mathbf{Z } = \{\mathbf{z }_1, \mathbf{z }_2, ..., \mathbf{z }_n\}$ of SARS-CoV-2 sequences. As illustrated in Fig. 2, the Auto-Encoder Network is a core module of AutoCoV. It is widely used for encoding input features to smaller dimensional features to reconstruct input data as much as possible [58, 59]. Given the input matrix $\mathbf{X }_{norm}$, an encoder network generates a hidden representation matrix $\mathbf{Z }$ and a decoder network generates the reconstructed input matrix $\tilde{\mathbf{X }}_{norm}$ via the following calculations:

- Single linear layer configuration: the layer $f(\cdot )$ takes $\mathbf{x }_i^T$ as input and generates $\mathbf{h }_i^T$ as output where $\mathbf{x }_i$ is the row vector of input $\mathbf{X }_{norm}$, i.e., *k*-mer frequency vector of *i*-th sequence. To avoid overfitting and improve generalization performances, batch normalization layer and dropout layer are applied on the layer as below:
  2 $$\begin{aligned} \mathbf{h }_i^T = f(\mathbf{x }_i^T) = \text {Dropout}(\sigma (\text {BN}(\mathbf{W }\mathbf{x }_i^T+\mathbf{b }))) \end{aligned}$$
  where $\mathbf{W }$ and $\mathbf{b }$ are a weight matrix and a bias vector for the linear layer, respectively, BN is the batch normalization layer, $\sigma (\cdot )$ is an activation function.
- Encoder network configuration: the goal of encoder network is generating the latent representation $\mathbf{z }_i^T$ from the input $\mathbf{x }_i^T$. Let the encoder network consists of *L* single linear layers. Then, $\mathbf{z }_i^T$ is calculated as below:
  3 $$\begin{aligned} \mathbf{z }_i^T = f^L \circ \cdot \cdot \cdot \circ f^2 \circ f^1(\mathbf{x }_i^T) \end{aligned}$$
  where the activation function $\sigma (\cdot )$ is *tanh* function in all layers and *L*-th layer has no dropout layer.
- Decoder network configuration: the goal of decoder network is to reconstruct the input data $\tilde{\mathbf{x }}_i^T$ from the latent representation $\mathbf{z }_i^T$. The structure of the decoder network is inversely same with that of the encoder network. Then, $\tilde{\mathbf{x }}_i^T$ is calculated as below:
  4 $$\begin{aligned} \tilde{\mathbf{x }}_i^T = f^{-1} \circ f^{-2} \circ \cdot \cdot \cdot \circ f^{-L} (\mathbf{z }_i^T) \end{aligned}$$
  where $f^{-l}$ is a corresponding decoding layer of *l*-th encoding layer $f^l$ and no tied weights are used for the network. The last layer $f^{-1}(\cdot )$ has no batch normalization and dropout layer.

To measure reconstruction performance, mean squared error (MSE) loss ${{\mathcal {L}}_{rec}}$ is calculated between $\mathbf{x }_i$ and $\tilde{\mathbf{x }}_i$ as below:

5 $$\begin{aligned} {{\mathcal {L}}_{rec}} = \frac{1}{d'}\sum _{j=1}^{d'}(x_{i,j} - {\tilde{x}}_{i,j})^2 \end{aligned}$$

### Classifier network

In general, standard Auto-Encoder Network generates latent representations $\mathbf{Z }$ and reconstructs input data well enough. In the case of SARS-CoV-2 sequences, however, it is hard to construct well-separated embedding spaces due to the high similarity of sequences. To achieve the distinct latent representations according to spatial and temporal patterns, we adopt an auxiliary Classifier Network on the Auto-Encoder Network. This auxiliary network will predict spatial labels for learning spatial patterns or temporal labels for learning temporal patterns. Like multi-task learning, this can be considered as a guide to learning the representations for spatial or temporal patterns, while reconstructing input data by the Auto-Encoder network. Given the latent representation $\mathbf{z }_i^T$ from the Auto-Encoder Network, a predicted class label $\tilde{\mathbf{y }}_i$ is calculated by *L* linear layers:

6 $$\begin{aligned} \tilde{\mathbf{y }}_i = \text {Softmax}(f^L \circ \cdot \cdot \cdot \circ f^2 \circ f^1(\mathbf{z }_i^T)) \end{aligned}$$

where the last layer $f^L$ has only fully connected layer. The Classifier Network is trained by minimizing the cross entropy loss ${{\mathcal {L}}}_{clf}$ as below:

7 $$\begin{aligned} {{\mathcal {L}}}_{clf} = - \sum _{c=1}^{C} y_{i,c} \text {log}({\tilde{y}}_{i,c}) \end{aligned}$$

where *C* is the number of classes and *c* is the index of class labels.

### Center loss

Cross entropy loss with softmax layer guides the model to learn features that distinguish classes. However, it is insufficient to learn compact representations of data belonging to the same class [60, 61]. For this reason, Wen et al. proposed a new loss function called center loss [60]. The basic concept of center loss is to minimize the distances between data within the same class. In this study, to learn compact embedding spaces for spatial or temporal patterns, label information of each sequence is utilized in the Center Loss module. Let ${\varvec{\mu }}_c$ denotes a mean vector of class *c*. The center loss for *i*-th sequence is measured as below:

8 $$\begin{aligned} {{\mathcal {L}}}_{ctr} = || z_i^T - {\varvec{\mu }}_{y_i} ||_2^2 \end{aligned}$$

where $y_i$ is the class of *i*th sequence. Using the center loss, model parameters are updated to bring latent representations closer to their mean vectors. Then, the mean vector for each class is re-calculated using the updated latent representations. That is, the latent representations and the mean vectors are updated simultaneously.

### Loss function and training model

For learning spatial and temporal patterns of SARS-CoV-2 effectively, AutoCoV utilize three loss functions: *MSE loss, cross entropy loss, and center loss*. Then, a total loss function for a sequence is formulated in the following equation:

9 $$\begin{aligned} {{\mathcal {L}}}_{total} = {{\mathcal {L}}_{rec}} + {{\mathcal {L}}_{clf}} +{{\mathcal {L}}_{ctr}} \end{aligned}$$

For mini-batch training, the total loss is normalized by the size of the mini-batch. We used Adam optimizer with two different learning rates for learning AutoCoV [62]. The first learning rate controls the overall network of the model except the center loss module. The second learning rate is used for the center loss module. In this study, to focus on generating compact and distinct latent representations, the learning rate for the center loss is larger than the overall learning rate.

## Supplementary Information

**Additional file 1:** Supplementary document containing basic information about SARS-CoV-2 and details information about materials, method, and additional results.

## Data Availability

The SARS-CoV-2 sequence can be downloaded from NCBI Virus (http://www.ncbi.nlm.nih.gov/genome/viruses/) and GISAID (http://www.gisaid.org). And the proposed model AutoCoV is freely available on GitHub (https://github.com/smaster7/AutoCoV).

## Abbreviations

SARS-CoV-2: Severe acute respiratory syndrome coronavirus 2
COVID-19: Coronavirus disease 2019
WHO: The World Health Organization
$R_0$: The basic reproductive number
NCBI: The National Center for Biotechnology Information
GISAID: Global initiative for sharing all influenza data
CNN: Convolutional neural network
NLP: Natural Language processing
DNN: Deep neural network
RNN: Recurrent neural network
2D: Two dimensional
MSE: Mean squared error
PCA: Principal component analysis
T-SNE: T-stochastic neighbor embedding
UMAP: Uniform manifold approximation and projection
CF: Classifier network
LHS: Label Homogeneous Score
MI: Mutual information
