# Supplementary material for: AutoCoV: tracking the early spread of COVID-19 in terms of the spatial and temporal patterns from embedding space by K-mer based deep learning
Source: BMC Bioinformatics. 2022 Apr 25;23(Suppl 3):149. doi: 10.1186/s12859-022-04679-x (PMC9036508; doi:10.1186/s12859-022-04679-x)
Supplement: Supplementary file 1 — Additional file 1: Supplementary document containing basic information about SARS-CoV-2 and details information about materials, method, and additional results. [file 12859_2022_4679_MOESM1_ESM.pdf]

Supplementary File

Preliminary for SARS-CoV-2

Severe acute respiratory syndrome coronavirus-2 (SARS-CoV-2) is a positive-sense single-stranded RNA virus belonging to the *Sarbecovirus* or lineage B subgenus in the *Betacoronavirus* genus in the *Coronaviridae* family in the *Nidovirales* order in the *Pisoniviricetes* class (Chan et al. 2020). The genome sequence is about 30,000 bases in length and each sequence is very similar, making it difficult to compare with each other. that it is difficult to compare each other. SARS-CoV-2 comprises of the untranslated region (UTR) at the front and back (5'-UTR, 3'-UTR) and open reading frames (ORFs): ORF1a, ORF1b, ORF2 (spike or S), ORF3a, ORF4 (envelope or E), ORF5 (membrane or M), ORF6, ORF7a, ORF7b, ORF8 and ORF9 (nucleocapsid or N) (Supplementary Fig. 1). The SARS-CoV-2 sequences were divided into six subclasses: S, L, V, G, GR and GH, which are clade information defined by the GISAID nomenclature system (Elbe and Buckland-Merrett 2017; Shu and McCauley 2017; Tang et al. 2020). GISAID defined six subclasses based on nine marker mutations for information obtained from the statistical distribution of genome distances in phylogenetic cluster (Han et al. 2019). The nine mutations defining six subclasses are described in Supplementary Table 1. In addition, SARA-CoV-2 has spatial and temporal features. For spatial characteristics, the sequence was divided by the continent or country from which it was collected. And for temporal characteristics, the sequence was classified as year-month(-day) based on the day it was collected, not the day it was sequencing.

SARS-CoV-2 spread from region to region over time and mutated in that spread. And the mutated virus spreads to other regions again. This spread pattern of viruses can be identified from a statistical perspective (Supplementary Fig. 2). Supplementary Fig. 2(a) shows a temporal spread pattern that how many different strains of the virus are collected. There are various causes of the situation and one of the most reasons may be the inter-regional movements over time. Supplementary Fig. 2(b) also shows a spatial spread pattern in which different subclasses may be mixed due to inter-regional diffusion.

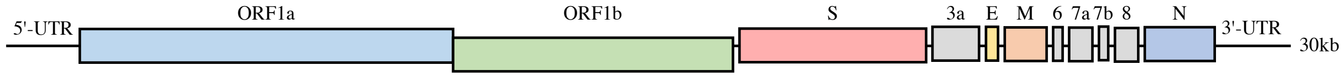

Supplementary Figure. 1: Genome organization of SARS-CoV-2. The sequence of SARS-CoV-2 is about 30 kb long and comprises of untranslated regions (5'-untranslated region and 3'-untranslated region) and various lengths of open reading frames (ORFs): ORF1a, ORF1b, ORF2 (spike or S), ORF3a, ORF4 (envelope or E), ORF5 (membrane or M), ORF6, ORF7a, ORF7b, ORF8 and ORF9 (nucleocapsid or N).

Supplementary Table 1: List of nine marker mutations defining six subclasses. The subclasses of SARS-CoV-2, defined by GISAID nomenclature system, are divided into six subclasses according to the combination of mutations. Each mutation is marked by a positional mutation based on the reference sequence. For example, C8782T means that C is mutated to T at position 8782.

Subclass	Variants
S	C8782T, T28144C
L	C241,C3037, A23403, C8782, G11083, G25563, G26144, T28144, G28882
V	G11083T, G26144T
G	C241T, C3037T, A23403G
GH	C241T, C3037T, A23403G, G25563T
GR	C241T, C3037T, A23403G, G28882A

Model Structures and Hyper-parameters

AutoCoV is the extended auto-encoder network by augmenting a classifier network and a center loss objective function. The auto-encoder network has four fully connected layers for each encoder and decoder part. The classifier network has two fully connected layers followed by the softmax function to predict class labels. Batch normalization and dropout techniques are utilized for improving the generalization performances of AutoCoV. With the model structure, hyper-parameters for mini-batch training are described in Supplementary Table 2. As mentioned in the main script, AutoCoV uses two learning rates: global and center loss. The global learning rate lr_{global} controls an overall network including auto-encoder and classifier network. The center loss learning rate lr_{cent}controls only the center loss module. To generate well clustered and separated latent spaces, lr_{cent} is larger than lr_{global}.

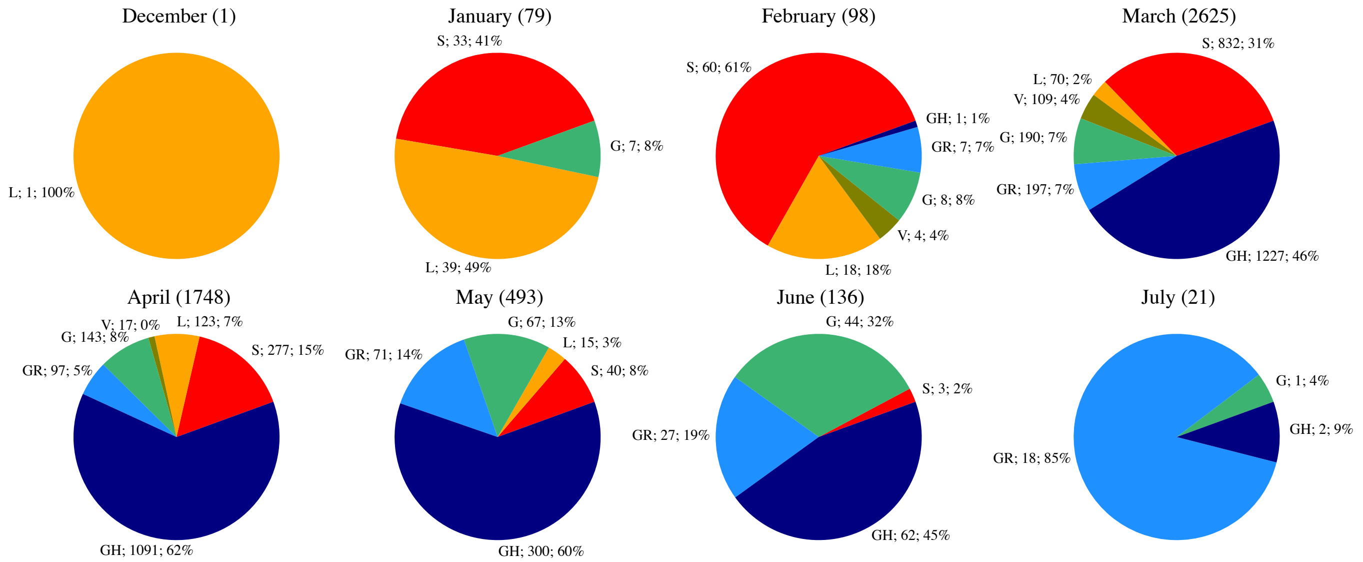

(a) Pie chart for each month

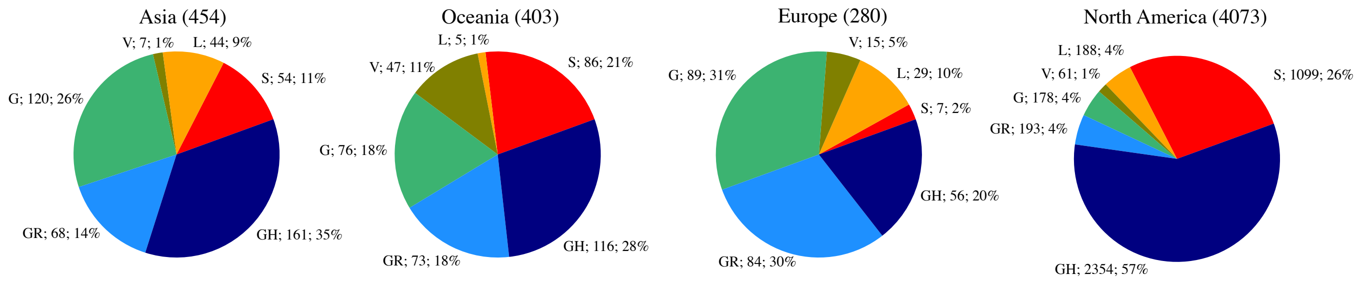

(b) Pie chart for each continent

Supplementary Figure. 2: Pie chart for the six subclasses of the SARS-CoV-2: (a) month (from December 2019 to July 2020) and (b) continent. The pie chart shows the number and percentage of subclasses in the total number of data for each title (Title (total number of data).

Supplementary Table 2: Model structure and hyper-parameters for AutoCoV

Hyper-parameters	Values
Auto-Encoder Network	[200 100 20 2 20 100 200]
Classifier Network	[2 16 NUM_CLASSES]
random seed	42
batch size	128
learning rate (global lr_{global})	0.01
learning rate (center loss lr_{cent})	0.5
dropout rate	0.2

Details of Baselines

Principal Component Analysis (PCA) (Pearson 1901)

The same as AutoCov, the k-mer frequency matrix produced by the preprocessing module is the input of PCA. The 2D embedding space is constructed by PC1 and PC2 of training data, and validation and test data are projected into the space. To compute PC1 and PC2 of the data, we utilized sklearn.decomposition.PCA from scikit learn library of Python. With default parameters, n_components is 2 and random_state is 42.

t-Stochastic Neighbor Embedding (t-SNE) (Maaten and Hinton 2008)

The input of t-SNE is the same as AutoCoV. Basically, t-SNE is not allowed out-of-sampling projection, i.e., new data cannot be projected into the trained space. As similar to (Kobak and Berens 2019), we first generated an embedding space by using the default setting of sklearn.manifold.TSNE from scikit learn library of Python. To project a new data point in the space, we obtained ten nearest train data points, called reference points, of a new data point in the input high-dimensional space. The position of the new data in the embedding space was calculated by the median location of the ten reference points in the embedding space.

Uniform Manifold Approximation and Projection (UMAP) (McInnes, Healy, and Melville 2018)

The input of UMAP is the same as AutoCoV. UMAP constructs a 2D embedding space using training data by general non-linear dimension reduction technique. It is a technique similar to t-SNE, but new data can be mapped to the trained space. As similar to PCA, we utilized publicly available UMAP library of Python (https://umap-learn.readthedocs.io/en/latest/).

dna2vec (Ng 2017)

Our setting followed the implementation done in dna2vec. Word2vec (Mikolov et al. 2013) of k-mers learns the representation of vec(k-mer1) + vec(k-mer2) $\approx$ vec(concat(k-mer1, k-mer2)). To capture such dependencies along different lengths k-mers, we sampled k from discrete uniform distribution Uniform(3, 8) during training. We averaged embeddings of all 6-mers in a sequence to get embedding of the sequence.

seq2vec (Kimothi et al. 2016)

We directly obtained embedding of a sequence using seq2vec. This approach is based on the work presented in doc2vec (Le and Mikolov 2014). Although seq2vec suggested different methods of preprocessing sequences and choosing hyper-parameters, our setting mainly followed that of dna2vec to highlight the difference between the models.

Seq2Seq+CF (Sutskever, Vinyals, and Le 2014)

We utilized Sequence to Sequence Networks (Seq2Seq) with Long Short Term Memory (LSTM) and a classifier network (CF). To fed biological sequences into the model, each sequence was divided into a set of 6-mers. To prevent the sentence from becoming too long, uninformative 6-mers were discarded by normalized entropy filtering with threshold 0.5. On the Seq2Seq framework, with hidden size 128, the encoder takes a sentence and learns the latent representation of the sequence. The decoder takes both the latent representation and a start token, and it reconstructs the input sequence. When training the model, the latent representation from the encoder is fed into the decoder every time step as well as each word of the target sequence. This lessens the burden on the model since it does not need to cram all the information of the input sequence into a single vector, making it more competent in handling long sequences. In addition, using the latent representation, a classifier network predicts the class label of the sequence.

BERT+CF (Devlin et al. 2018)

BERT model with Classifier module was used to obtain latent representations of biological sequences. We used a similar preprocessing method to what we have used in Seq2Seq model. However, for BERT model, we chose the number of 6-mer types to be kept instead of the threshold of entropy value. Out of 4,096 6-mers types, thirty 6-mer types with the highest entropy values were chosen and kept from the sequence. For both spatial and temporal patterns tasks, we used the same training procedure and BERT/Pretraining/Classifier modules only except for the output layer for Classifier module. Our BERT model consisted of 6 Transformer blocks, hidden size 768, and 12 self-attention heads. We followed BERT Masked Language Model procedure for pretraining. The next sentence prediction was not applicable in our case. After pretraining, we trained Classifier module with BERT parameters fixed. Classifier module consisted of a max pooling layer and 2 linear layers. The last hidden layer with 240 dimensions was selected as a latent representation of the input sequences.

Details of Evaluation Metrics

Label Homogeneous Score (LHS)

To simultaneously consider subclass pattern, this score is computed for each subclass and averaged across all subclass labels. For the sequences belonging to the specific subclass c_i, T centroids in the embedding space are obtained where T is the number of class labels $t_1, t_2, ..., t_T$. A predicted label of each sequence is assigned by the nearest centroid. With the true labels of the sequences, denoted as Y, and the predicted labels, denoted as $\tilde{Y}$, LHS of the embedding space is measured as below:

$$LHS = \frac{1}{C} \sum_{c_i}^{C} \phi(Y, \tilde{Y} | c_i) \tag{1}$$

where ϕ is a function from the sklearn.metrics.homogeneity_score.

Mutual Information Score (MI)

The axes of the embedding space are divided into 10 equal spaces. Then, the space is changed into the grid space with 100 cells. For each cell, the ratio of each class label y is measured and used as the probability $P_{\{\text{Dim1, Dim2}\},y}$. Using the probabilities of all cells, MI is calculated as below:

$$MI = \sum_{\text{Dim1,Dim2}} \sum_{y} P_{\{\text{Dim1, Dim2}\},y} \log \frac{P_{\{\text{Dim1, Dim2}\},y}}{P_{\{\text{Dim1, Dim2}\}} P_y} \tag{2}$$

where $P_{\{\text{Dim1, Dim2}\}}$ is the probability of how many sequences are located in the cell, and P_y is the probability of how many sequences are belonging to the class y.

F_1 score

Based on the embedding space generated by training data, a k-nearest neighbor classifier is trained (# of neighbors = 10). Then, the predicted labels of validation/test data are assigned by the classifiers. To predict the labels, sklearn.neighbors.KNeighborsClassifier is utilized and the F_1 score is measured by sklearn.metrics.f1_score with weighted average option.

Preprocessing of GISAID dataset

The Global Initiative for All Influenza Data Sharing (GISAID) is a global non-profit organization that provides genomic data for all influenza viruses and the coronavirus that causes COVID-19. Among 61,210 SARS-CoV-2 sequences downloaded from the GISAID by July 2020, we excluded low-quality sequences such as sequence contains lowercase nucleotide, exists more than 100 N's, includes mutation other than nine marker mutation or without spatial or temporal label information. Therefore, we used 23,979 sequences as external data. And then we conducted 10 times stratified sampling to preserve the ratio of each label in the spatial and temporal class of the NCBI train set. The k-mer frequency vector of the unannotated sequence was constructed using a portion similar to the front 50-mer of the ORF1a gene to a portion similar to the terminal 50-mer of the N gene, based on the reference sequence. Here we allowed more than 80% similarity. And then only the significant k-mers used in train data were selected to construct the frequency matrix of unannotated sequences.

Additional Experiment Results

Patterns on Train/Validation/Test

In this section, we will show spatial and temporal patterns of train and validation data used for the comparison results in main script (Supplementary Fig. 4 and 5). A subclass pattern on the space where specific pattern is learned is also represented (Supplementary Fig. 6).

Various sizes of k in k-mer

Since we encoded the sequence using k-mer, the size of k in the k-mer would result in a performance difference. Hence, we investigated how the performance varies depending on the size of k in terms of three evaluation metrics: LHS, MI and F_1. When the size of k ranges from 1 to 7, Supplementary Fig. 3 (a) and (b) show that the results are similar with k from 3 to 6 for all evaluation metrics in both spatial and temporal patterns. Among them, when the size of k is 3 or 6, that is, k-mer consists of three or six nucleotides, the k-mer reflects biological prior knowledge such as amino acid (also called a codon) or dicodon (two amino acids). Therefore, in this study, we utilized k as 6 to cover the performance aspect of the model and the aspect of biological prior knowledge.

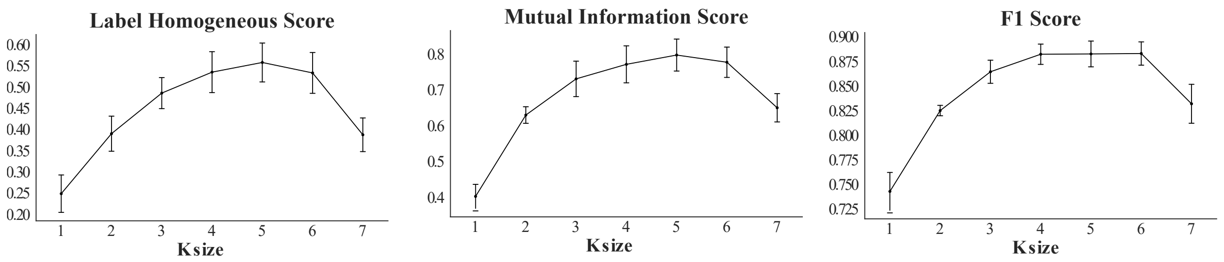

(a) Spatial

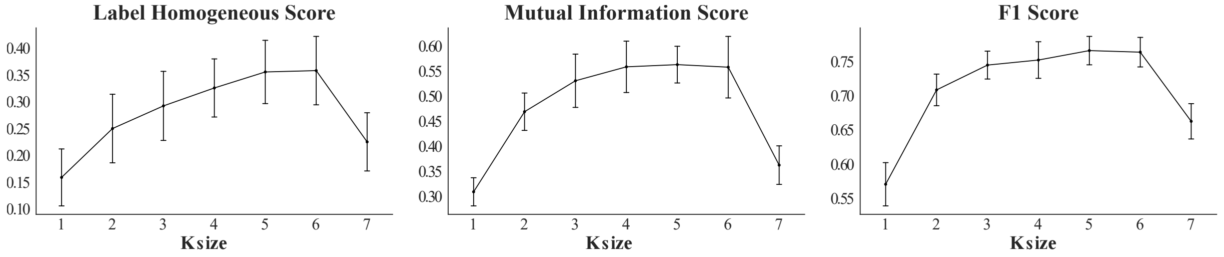

(b) Temporal

Supplementary Figure. 3: Performance of AutoCoV with various sizes of k in k-mer. The x-axis of the plot represents the size of k from 1 to 7 and the y-axis represents the result of the evaluation metric: LHS (Label Homogeneous Score, left), MI (Mutual Information Score, middle) and F_1 Score (right) in (a) spatial and (b) temporal patterns, respectively. The error bar at each point represents the standard deviation.

Supplementary Table 3: Ablation study results (mean $\pm$ std). The two unsupervised methods (AE, AE+BN) and six supervised methods (AE+CL, AE+BN+CL, AE+CF, AE+BN+CF, AE+CL+CF, AutoCoV) are compared, and the bold values represent the best performance among them. In both patterns, AutoCoV outperforms the ablation experiments in all three metrics.

Pattern	Metric	Unsupervised		Supervised					
		AE	AE+BN	AE+CL	AE+BN+CL	AE+CF	AE+BN+CF	AE+CL+CF	AutoCoV
Spatial	LHS	0.194 ± 0.066	0.270 ± 0.059	0.265 ± 0.056	0.274 ± 0.060	0.440 ± 0.054	0.523 ± 0.040	0.483 ± 0.047	**0.529 ± 0.051**
	MI	0.265 ± 0.058	0.346 ± 0.045	0.358 ± 0.026	0.409 ± 0.051	0.592 ± 0.062	0.751 ± 0.049	0.672 ± 0.063	**0.773 ± 0.045**
	F_1	0.769 ± 0.021	0.792 ± 0.017	0.809 ± 0.018	0.799 ± 0.022	0.860 ± 0.015	0.879 ± 0.012	0.868 ± 0.013	**0.881 ± 0.012**
Temporal	LHS	0.102 ± 0.051	0.136 ± 0.067	0.133 ± 0.055	0.157 ± 0.060	0.242 ± 0.053	0.348 ± 0.082	0.312 ± 0.071	**0.355 ± 0.067**
	MI	0.119 ± 0.025	0.179 ± 0.024	0.144 ± 0.042	0.198 ± 0.034	0.362 ± 0.081	0.542 ± 0.051	0.452 ± 0.067	**0.554 ± 0.065**
	F_1	0.552 ± 0.077	0.655 ± 0.019	0.673 ± 0.024	0.665 ± 0.017	0.707 ± 0.037	**0.761 ± 0.028**	0.736 ± 0.031	**0.761 ± 0.023**

Ablation Study

AutoCoV consists of modules such as Classifier Network (CF), Batch Normalization (BN), and Center Loss (CL). To measure the contribution of each module, we conducted ablation experiments to investigate the contribution of various modules on learning spatial and temporal patterns of SARS-CoV-2. Using Auto-Encoder Networks (AE) as a base, we designed eight combinations of the three modules and constructed the eight different models.

Supplementary Table 3 shows the results of different combinations in the three metrics: LHS, MI, KNN. The following conclusions were derived from the results:

1. Classifier Network was the most crucial module of AutoCoV. Augmenting the module can bring significant improvements in all associated combinations.

2. The combination of classifier and center loss, i.e., AE+CL+CF shows better performances than AE+CF but not than AE+BN+CF. With augmenting batch normalization on AE+CL+CF, i.e., AutoCoV, it improved the performances by generating synergies between the two modules.

3. Batch Normalization can affect the robustness of the model performance. In general, relatively lower standard deviation is achieved with Batch Normalization.

4. Interestingly, the base auto-encoder using k-mer frequency with information theoretic filtering showed better performances than most of the other baselines in Table 2 in the main script. It can be seen that the combination of auto-encoder and k-mer was an efficient approach to learning latent representations of SARS-CoV-2.

High-dimensional embedding for dna2vec and seq2vec

Instead of embedding 6-mers into two-dimensional vectors, we performed dna2vec and seq2vec with high-dimensional embedding vectors ($d = 100$). As the dimension increases, it is not possible to directly construct a patterns space in two dimensions, but it may be possible to learn more various information. Supplementary Fig. 7 and 8 shows the 2D embedding space of each pattern generated by UMAP. As the latent dimension of dna2vec increases, although it still did not learn the spatial and temporal patterns well, it was shown that the clustered sequences spread in the space. In contrast, in the case of seq2vec, there was no significant difference even if the dimension increased.

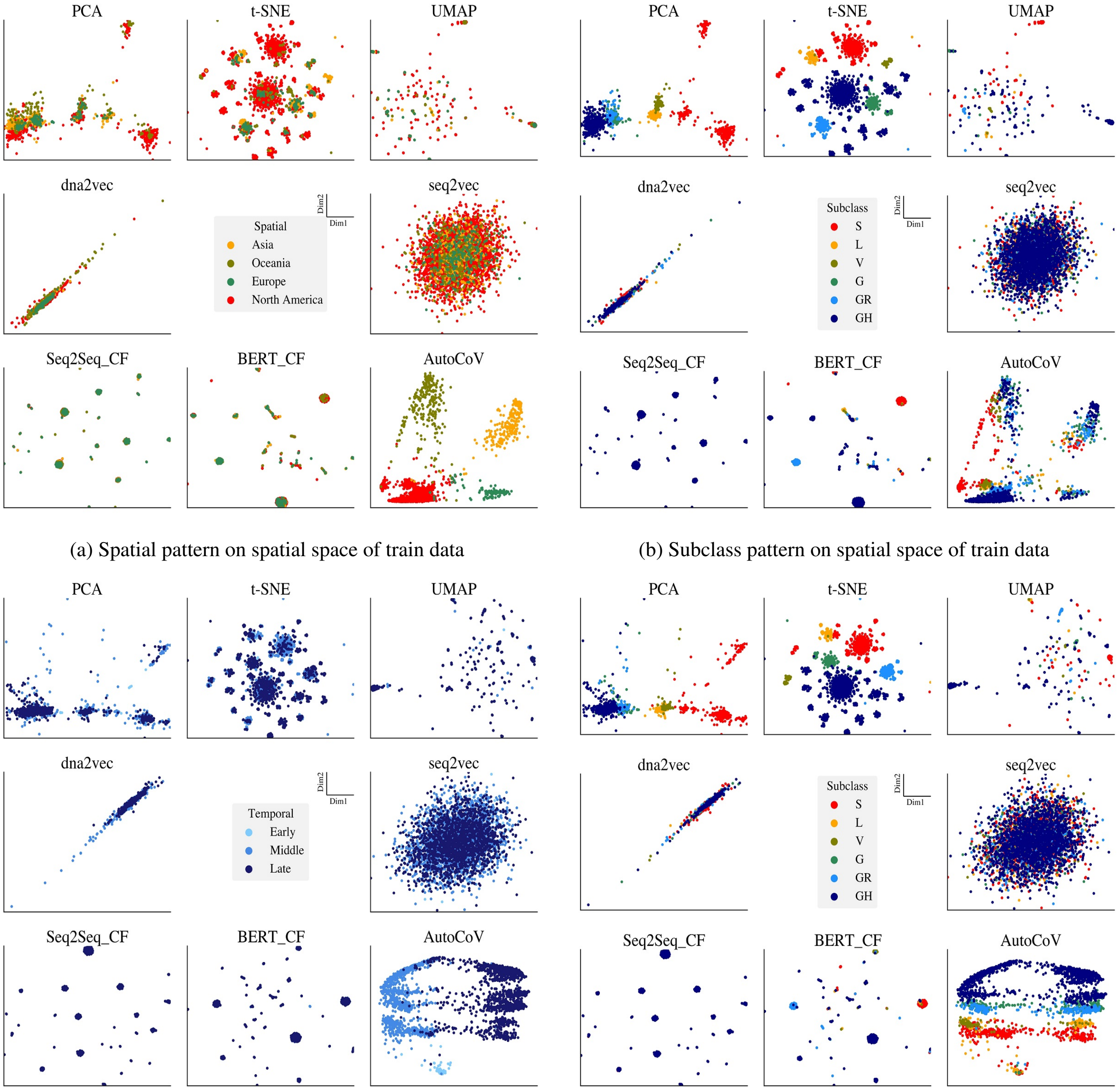

(a) Spatial pattern on spatial space of train data

(b) Subclass pattern on spatial space of train data

(c) Temporal pattern on temporal space of train data

(d) Subclass pattern on temporal space of train data

Supplementary Figure. 4: Comparison results on train data. 2D embedding spaces of baselines and AutoCoV are illustrated as (a) the spatial pattern, and (b) the subclass pattern on spatial spaces. As similar, (c) the temporal pattern, and (d) the subclass pattern on temporal spaces. The data split of median fold by AutoCoV was used for the figures.

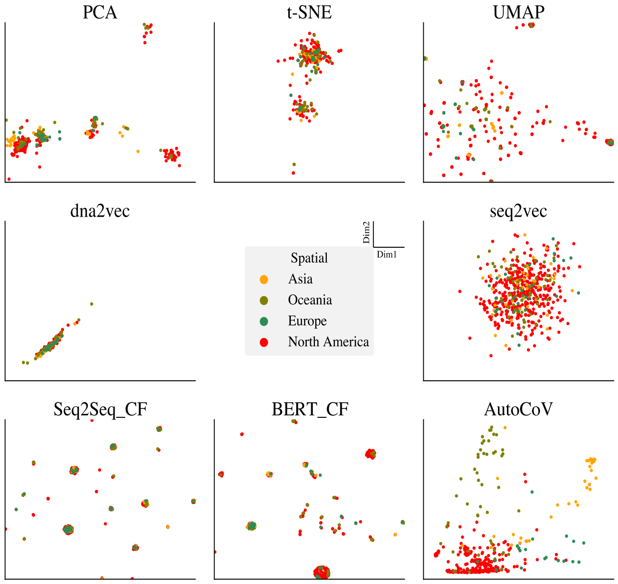

(a) Subclass pattern on spatial space of validation data

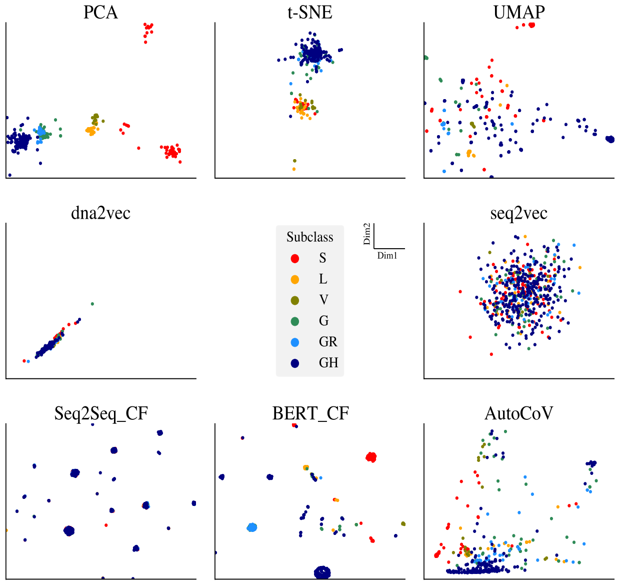

(b) Subclass pattern on spatial space of validation data

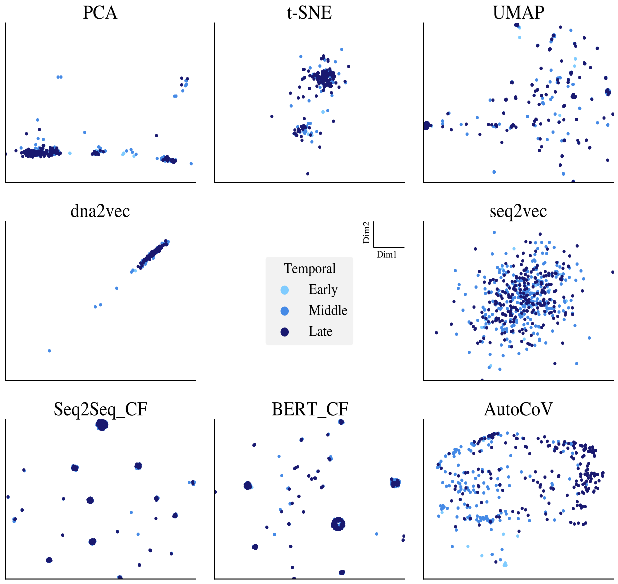

(c) Temporal pattern on temporal space of validation data

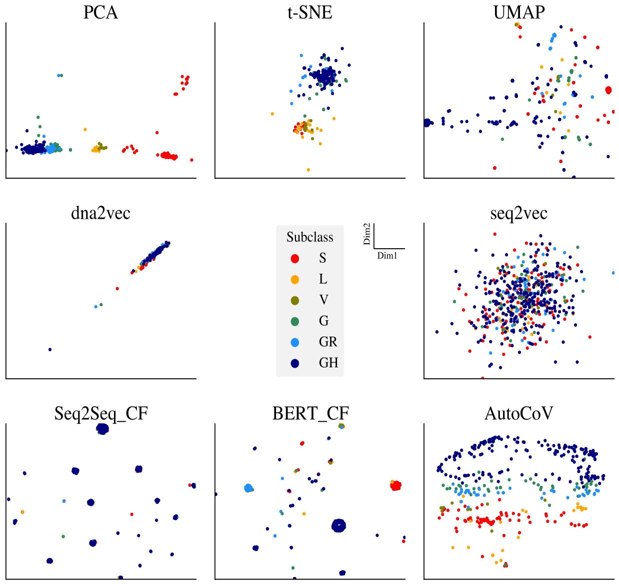

(d) Subclass pattern on temporal space of validation data

Supplementary Figure. 5: Comparison results on validation data. 2D embedding spaces of baselines and AutoCoV are illustrated as (a) the spatial pattern, and (b) the subclass pattern on spatial spaces. As similar, (c) the temporal pattern, and (d) the subclass pattern on temporal spaces. The data split of median fold by AutoCoV was used for the figures.

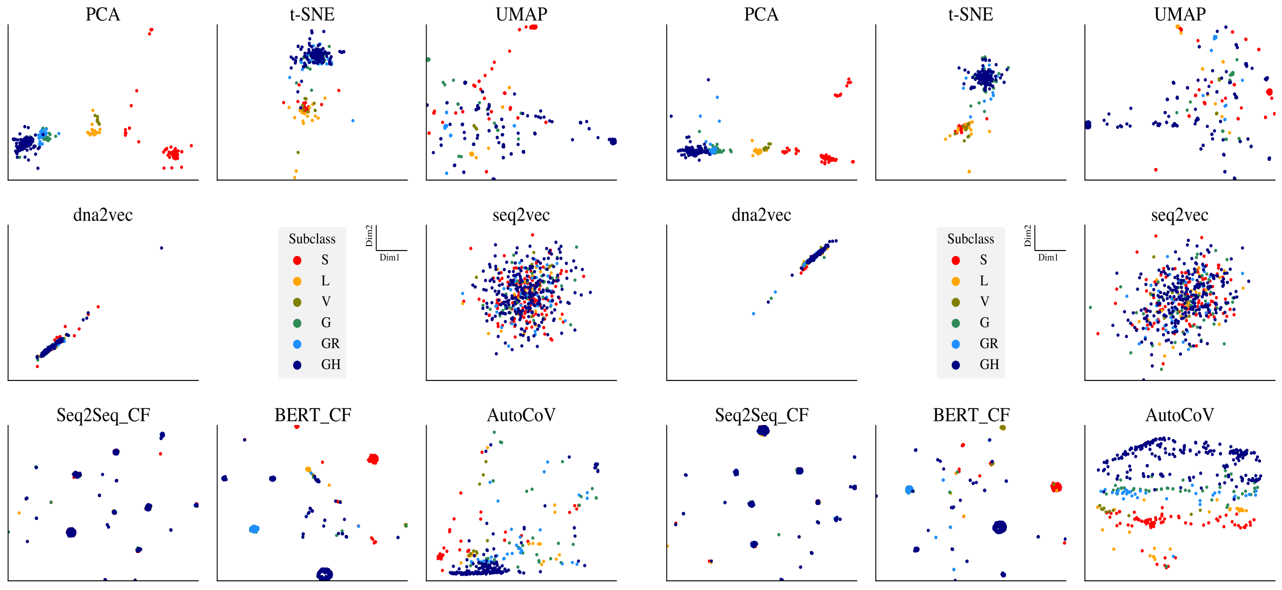

(a) Spatial pattern space

(b) Temporal pattern space

Supplementary Figure. 6: Subclass patterns of test data on (a) the spatial pattern, and (b) the temporal pattern space. The spaces are same as Fig. 3 in main script.

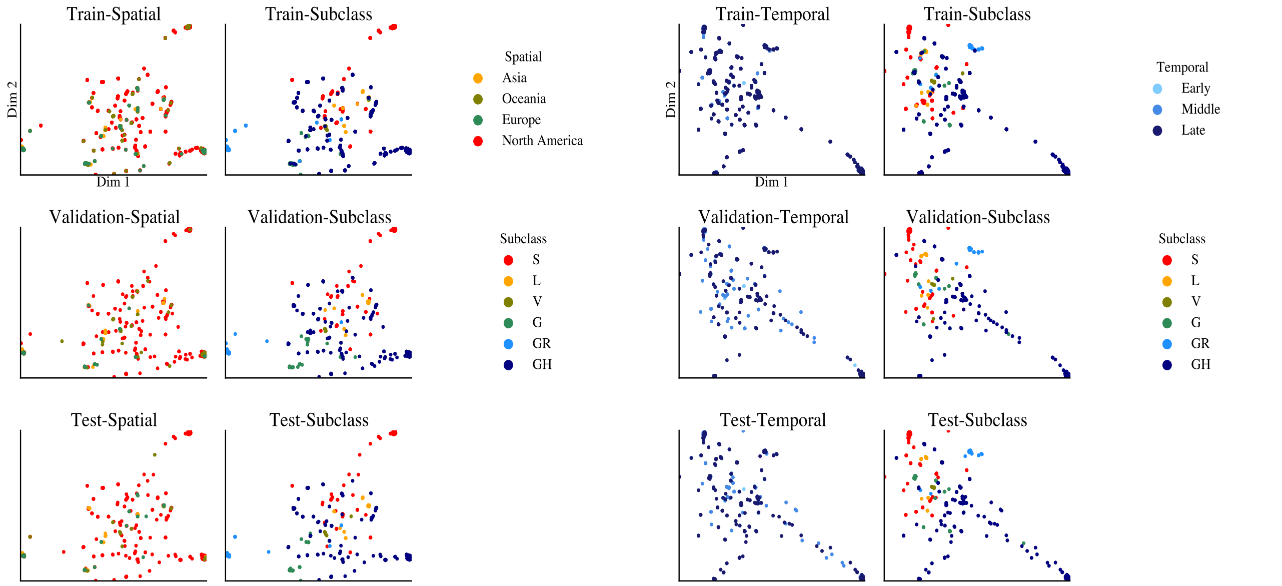

(a) Spatial pattern space

(b) Temporal pattern space

Supplementary Figure. 7: dna2vec with dimension of latent representation = 100. Instead of dim = 2, we generated an embedding of a sequence into the high-dimensional vector. 2D embedding is performed by UMAP.

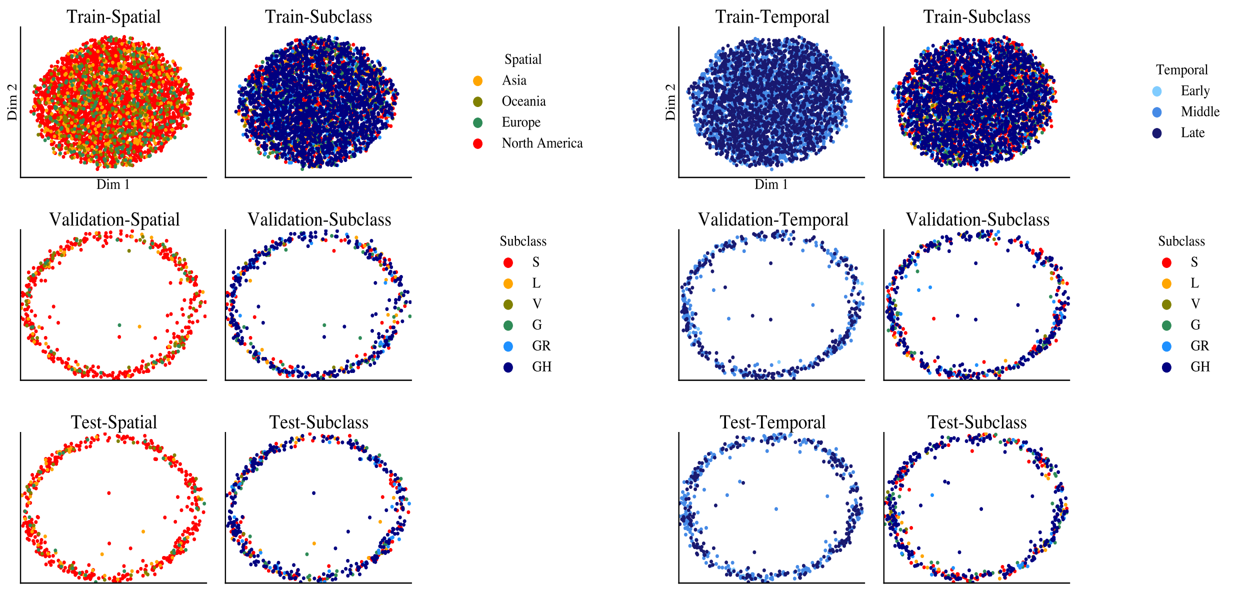

(a) Spatial pattern space

(b) Temporal pattern space

Supplementary Figure. 8: seq2vec with dimension of latent representation = 100. Instead of dim = 2, we generated an embedding of a sequence into the high-dimensional vector. 2D embedding is performed by UMAP.

References

Chan, J. F.-W.; Kok, K.-H.; Zhu, Z.; Chu, H.; To, K. K.-W.; Yuan, S.; and Yuen, K.-Y. 2020. Genomic characterization of the 2019 novel human-pathogenic coronavirus isolated from a patient with atypical pneumonia after visiting Wuhan. *Emerging microbes & infections* 9(1): 221–236.

Devlin, J.; Chang, M.-W.; Lee, K.; and Toutanova, K. 2018. Bert: Pre-training of deep bidirectional transformers for language understanding. *arXiv preprint arXiv:1810.04805* .

Elbe, S.; and Buckland-Merrett, G. 2017. Data, disease and diplomacy: GISAID's innovative contribution to global health. *Global Challenges* 1(1): 33–46.

Han, A. X.; Parker, E.; Scholer, F.; Maurer-Stroh, S.; and Russell, C. A. 2019. Phylogenetic clustering by linear integer programming (PhyCLIP). *Molecular biology and evolution* 36(7): 1580–1595.

Kimothi, D.; Soni, A.; Biyani, P.; and Hogan, J. M. 2016. Distributed representations for biological sequence analysis. *arXiv preprint arXiv:1608.05949* .

Kobak, D.; and Berens, P. 2019. The art of using t-SNE for single-cell transcriptomics. *Nature Communications* 10(1): 1–14.

Le, Q.; and Mikolov, T. 2014. Distributed representations of sentences and documents. In *International Conference on Machine Learning*, 1188–1196.

Maaten, L. v. d.; and Hinton, G. 2008. Visualizing data using t-SNE. *Journal of Machine Learning Research* 9(Nov): 2579–2605.

McInnes, L.; Healy, J.; and Melville, J. 2018. Umap: Uniform manifold approximation and projection for dimension reduction. *arXiv preprint arXiv:1802.03426* .

Mikolov, T.; Chen, K.; Corrado, G.; and Dean, J. 2013. Efficient estimation of word representations in vector space. *arXiv preprint arXiv:1301.3781* .

Ng, P. 2017. dna2vec: Consistent vector representations of variable-length k-mers. *arXiv preprint arXiv:1701.06279* .

Pearson, K. 1901. LIII. On lines and planes of closest fit to systems of points in space. *The London, Edinburgh, and Dublin Philosophical Magazine and Journal of Science* 2(11): 559–572.

Shu, Y.; and McCauley, J. 2017. GISAID: Global initiative on sharing all influenza data–from vision to reality. *Eurosurveillance* 22(13): 30494.

Sutskever, I.; Vinyals, O.; and Le, Q. V. 2014. Sequence to sequence learning with neural networks. In *Advances in neural information processing systems*, 3104–3112.

Tang, X.; Wu, C.; Li, X.; Song, Y.; Yao, X.; Wu, X.; Duan, Y.; Zhang, H.; Wang, Y.; Qian, Z.; et al. 2020. On the origin and continuing evolution of SARS-CoV-2. *National Science Review* .
